# The novel taxon *Lacunimicrobium album* gen. nov., sp. nov. is constituted by three planctomycetotal strains isolated from freshwater habitats in Northern Germany

**DOI:** 10.1038/s41598-025-27459-y

**Published:** 2025-11-21

**Authors:** Moses Kabuu, Gaurav Kumar, Tom Haufschild, Jonathan Hammer, Christian Jogler, Nicolai Kallscheuer

**Affiliations:** 1 Department of Microbial Interactions, Institute for Microbiology, Friedrich Schiller University, Jena, Germany; 2 Cluster of Excellence Balance of the Microverse, Friedrich Schiller University, Jena, Germany

**Keywords:** *Planctomycetota*, Limnic bacteria, Schlesner strain collection, Budding bacteria, Ecology, Ecology, Evolution, Genetics, Microbiology

## Abstract

The majority of characterized members of the phylum *Planctomycetota* were isolated from marine habitats. To expand the open collection of limnic planctomycetes, we here characterize three isolates from the strain collection of Heinz Schlesner (Kiel University), a pioneer in the research on budding planctomycetes and other bacteria with uncommon morphologies. The three strains SH203, SH248^T^and SH280 were isolated from two municipal ponds in Kiel and from a nature reserve close to a gypsum mine in the surroundings of Hamburg, Northern Germany. The three isolates share an identical 16S rRNA gene sequence over the analyzed sequence of ca. 1300 bp. Cells are pear-shaped, motile, non-pigmented and show the typical asymmetrical division mode through polar budding observed in most other members of the family *Planctomycetaceae*. Phylogenetic analyses identified the three described species of the genus *Rubinisphaera* as the current closest relatives. The comparison of five phylogenetic markers along with differences in phenotypic and genomic features together delineate the novel strains from the genus *Rubinisphaera*. Hence, we introduce *Lacunimicrobium album* gen. nov., sp. nov., a novel taxon in the family *Planctomycetaceae*. The novel species is represented by the type strain SH248^T^(= CECT 30703^T^ = STH01007^T^; the STH number refers to the Jena Microbial Resource Collection JMRC). It has a heterotrophic, mesophilic and neutrophilic profile and a genome with a length of 5.42 Mbp and a DNA G + C content of 54.6%.

**Supplementary Information:**

The online version contains supplementary material available at 10.1038/s41598-025-27459-y.

## Introduction

Bacteria inhabit diverse environments and play a vital role in nature. Despite the high number of bacteria predicted to live on Earth, only about 2% are believed to have been cultured, leaving 98% of the suspected diversity undiscovered^1^. For many decades, members of the ubiquitous bacterial phylum *Planctomycetota* were considered unculturable up until the 1970s when the first pure cultures were reported^2^. From the 1980s to early 2000s, a large number of strains have been isolated from a variety of habitats in a sampling campaign led by Heinz Schlesner from Kiel University, Germany^3–5^. He isolated and analyzed strains that show uncommon morphologies or divide by budding, which resulted in a collection of ca. 500 strains, half of which turned out to be yet uncharacterized members of the phylum *Planctomycetota*. Today, more than four decades later, more than 140 planctomycetal species are available from public strain collections. However, several families of this phylum are still undersampled^6^.

Planctomycetes follow an uncommon and uncharacterized asymmetric cell division mechanism (“budding”) which is characterized by the absence of most of the otherwise essential cell division proteins, including FtsZ, the hallmark protein for division by binary fission^7–11^. Their cell walls contain peptidoglycan^12,13^, however, many of the otherwise essential peptidoglycan biosynthetic genes are dispensable in the limnic model species *Planctopirus limnophila* ^14^. In addition, phylum members show further variations to the Gram-negative cell plan which include, for instance, condensed DNA and invaginations of the cytoplasmic membrane that create an enlarged periplasmic space.

Many planctomycetal isolates have been shown to utilize high molecular-weight sugars derived from nutrient-rich surfaces of macroscopic phototrophs, mostly in aquatic ecosystems^15,16^. Abundances of up to 80% in bacterial communities on marine surfaces remain astounding considering their slow to moderate growth compared to other heterotrophic microorganisms in the same ecological niche^17,18^.

Members of the phylum *Planctomycetota* fascinate in several ways. Their uncommon cell biology along with high proportions of genes of unknown function, potential biotechnological applications as source of novel bioactive compounds are only a few of the topics under study^19–23^. Most strikingly, recently even planctomycetal strains were described that feed on other bacteria, incorporating them by a phagocytosis-like mechanism^24,25^.

The current research on the bacterial phylum is supported by automated genome mining^26,27^ and the optimization of genetic tools applicable in model strains^28,29^. In addition to more detailed analyses on model species, the isolation and characterization of novel, in particular limnic isolates should urgently be continued since the phylum is still undersampled with a strong bias towards marine habitats. For example, we obtained strains from marine surface waters^30^, marine sediments^31,32^, marine phototrophs such as micro- and macro algae^33^, from marine volcanic areas^34,35^ and from surfaces, both biotic^36,37^ and abiotic^38,39^. Few planctomycetal strains were isolated from marine animals such as sponges^40,41^or jellyfish^42^. In contrast, in our hands far fewer strains were obtained from brackish or limnic habitats such as ponds^43^, rivers^44^, wastewater^45^or freshwater sponges^46^. Thus, the isolation and characterization of novel, in particular limnic isolates should urgently be continued^47^.

Hence, we here describe three limnic strains from the Schlesner strain collection, SH203, SH248^T^, and SH280, that were isolated from two different ponds in Kiel and water from a gypsum mine.

## Materials and methods

### Strain isolation and cultivation conditions

The strains SH203, SH248^T^, and SH280 were isolated by Heinz Schlesner from a gypsum mine at Klein Nordende (exact location: 53.720873, 9.681402), from a campus pond at Kiel University (exact location: 54.326580, 10.122780), and from Schrevenpark pond in Kiel (exact location: 54.344637, 10.112395), respectively (all sampling spots are located in Northern Germany and the actual isolation was performed in the 1980s)^48^. The sampling sites had a pH of 7.55, 7.90, and 7.60, respectively^3^. The media M13a and M13(3x) were used for the initial inoculation from the cryo stocks^3,48,49^. However, the strains showed faster growth in M1H NAG AFW (synonym: limnic M1 medium) which was used for further cultivation. To prepare one liter of M1H NAG AFW medium, 0.53 mg NH_4_Cl, 1.4 mg KH_2_PO_4_, 10 mg KNO_3_, 49.3 mg MgSO_4_× 7 H_2_O, 14.7 mg CaCl_2_× 2 H_2_O, 25 mg CaCO_3_, 25 mg NaHCO_3_, 2.38 g 4-(2-hydroxyethyl)-1-piperazineethanesulfonic acid (HEPES), 0.25 g peptone (Bacto), 0.25 g yeast extract (Bacto), and 20 mL sterile-filtered Hutner’s basal salt solution were mixed in a final volume of 973 mL double distilled water. The medium pH was adjusted to 7.0 using 5 M KOH and the solution was autoclaved for 20 min at 121 °C. After cooling, the medium was aseptically supplemented with 1 mL of 25% (w/v) glucose, 20 mL of 50 g/L *N*-acetyl glucosamine (NAG) as carbon and nitrogen source, 5 mL of vitamin solution, and 1 mL of trace element solution. Hutner’s basal salt solution was prepared by first dissolving 10 g nitrilotriacetic acid (NTA) in 700 mL double distilled water and adjusting the pH to 7.2 using 5 M KOH. This was followed by the addition of 29.7 g MgSO_4_× 7 H_2_O, 3.34 g CaCl_2_× 2 H_2_O, 0.01267 g Na_2_MoO_4_× 2 H_2_O, 0.099 g FeSO_4_× 7 H_2_O and 50 mL metal salt solution 44. The solution was filled up to 1 L, filter-sterilized, and stored at 4 °C. Metal salt solution 44 consisted of 250 mg/L Na_2_-EDTA, 1095 mg/L ZnSO_4_× 7 H_2_O, 500 mg/L FeSO_4_× 7 H_2_O, 154 mg/L MnSO_4_x H_2_O, 39.5 mg/L CuSO_4_× 5 H_2_O, 20.3 mg/L CoCl_2_× 6 H_2_O and 17.7 mg/L Na_2_B_4_O_7_× 10 H_2_O. In the initial step, EDTA was dissolved with a few drops of concentrated H_2_SO_4_added to retard the precipitation of heavy metal ions. Metal salt solution 44 was sterile-filtered and stored at 4 °C. The vitamin solution contained per liter: 10 mg *p*-aminobenzoic acid, 4 mg biotin, 20 mg pyridoxine hydrochloride, 10 mg thiamine hydrochloride, 10 mg calcium pantothenate, 4 mg folic acid, 10 mg riboflavin, 10 mg nicotinamide, and 4 mg vitamin B_12_. *p*-Aminobenzoic acid was dissolved first to avoid solubility problems of the other vitamins, and the final solution was sterile-filtered and stored in the dark at 4 °C. The trace element solution containing 1.5 g/L Na-nitrilotriacetate, 500 mg/L MnSO_4_x H_2_O, 100 mg/L FeSO_4_× 7 H_2_O, 100 mg/L Co(NO_3_)_2_× 6 H_2_O, 100 mg/L ZnCl_2_, 50 mg/L NiCl_2_× 6 H_2_O, 50 mg/L H_2_SeO_3_, 10 mg/L CuSO_4_× 5 H2O, 10 mg/L AlK(SO_4_)_2_× 12 H_2_O, 10 mg/L H_3_BO_3_, 10 mg/L NaMoO_4_× 2 H_2_O and 10 mg/L Na_2_WO_4_× 2 H_2_O was sterile-filtered and stored in the dark at 4 °C. The three isolated strains were cultivated at 28 °C with slight agitation (90 rpm). Solidified M1H NAG AFW medium was prepared by the addition of 15 g/L agar that was washed three times with double distilled H_2_O, autoclaved separately in 200 mL ddH_2_O and mixed with the medium solution shortly before pouring of the plates. The other medium components were mixed and autoclaved in a total volume of 800 mL to yield a volume of 1 L after addition of the autoclaved agar.

### Light microscopy and cell size determination

The colony morphology was observed using Binocular microscopy. Phase-contrast (PhC) and Differential interference contrast (DIC) analyses were performed employing a Nikon Eclipse Ti2 using the NIS Elements software (Version 5.42.03) and a Nikon Plan Apo λ 100x oil objective as previously described^50^. Briefly, strains SH203, SH248^T^, and SH280 were grown to the half-maximal optical density at 600 nm (OD_600_) estimated to be 1.1, 1.3, and 0.7, respectively. 2 µL of the culture were immobilized on glass slides using a 1% (w/v) agarose cushion and the cover glass was sealed with 33% (w/w) paraffin to minimize evaporation^51^. The cell size analysis was conducted as previously described^45^. Image analysis was performed with BacStalk Version 1.8^52^ and visualization with SuperPlotsOfData^53^. In total, three biological replicates with 150 individual cells each were analyzed per strain.

### Physiological analyses

To determine the temperature optimum for growth, 1:4000 dilutions of actively growing cultures of the three strains were used for the inoculation of M1H NAG AFW agar in duplicates. These cultures were incubated at different temperatures ranging from 4 to 42 °C. For determination of the pH optimum for growth, liquid M1H NAG AFW was prepared using various buffers at different pH values. For cultivations at pH 7.0, 7.5, and 8.0, 100 mM HEPES was used. For cultivation at pH 5.0 and 6.0, HEPES was substituted with 100 mM 2-(*N*-morpholino)ethanesulfonic acid (MES) while for the cultivation at pH 9.0 and 10.0 100 mM *N*-cyclohexyl-2-aminoethane sulfonic acid (CHES) was used. The medium at pH 9.0 and 10.0 was filter-sterilized to avoid precipitation. The cultivation was carried out in an Epoch 2 Microplate Spectrophotometer (Agilent, Waldbronn, Germany) with the Gen5 software at 28 °C and the data was analyzed using Graphpad Prism 9.

Potential auxotrophies for vitamins were tested by cultivating the strains in M1H NAG AFW medium buffered with 100 mM HEPES at pH 7.0 at four different conditions: (1) complete medium, (2) medium without vitamin solution, (3) medium with a modified vitamin solution only lacking vitamin B_12_, (4) medium without vitamin solution, but only supplemented with vitamin B_12_. A blank (medium without cells) was used as a negative control. To avoid carryover of vitamin B_12_from the pre-cultures, the cell pellet was washed five times with M1H NAG AFW medium lacking vitamin B_12_prior to inoculation of the main culture. Cells were collected by centrifugation at 4,000 rpm for 5 min at 4 °C in each washing step. The cell pellet was then resuspended in the same medium and 100 µL was used to inoculate 2 mL in the different medium formulations stated above. The initial OD_600_was recorded, and the cultures were incubated at 28 °C with agitation (140 rpm). The OD_600_values were recorded for 7 days until the maximum OD_600_was reached. Anaerobic growth was examined on M1H NAG AFW plates which were incubated at 28 °C in an anaerobic jar at 0.02% or 0.22 µmol/L O_2_concentration as well as in liquid cultures purged with dinitrogen gas.

### Genomic DNA isolation and sequencing

For genomic DNA extraction, cell pellets of bacterial cultures growing at mid-exponential phase were harvested by centrifugation at 16,000 g for 2 min and stored at-20 °C. The genomic DNA (for long-read sequencing) was isolated using the Wizard HMW DNA Extraction Kit (Promega) following the manufacturer’s instructions. Oxford Nanopore sequencing, genome assembly and polishing with Illumina reads was performed as previously described^45^.

### Initial amplification of the 16S rRNA gene and phylogenetic analyses

The strains were initially identified based on their 16S rRNA gene that was amplified by PCR based on a standardized workflow^54^ and sequenced at Macrogen Europe (Amsterdam, The Netherlands). The current closest neighbors were identified via nucleotide Blast. For the more detailed analyses, the 16S rRNA gene sequence of the isolates and all characterized members of the phylum (as of July 2025) were aligned with ClustalW^55^. The alignment was used to calculate a 16S rRNA similarity matrix with TaxonDC^56^. A 16S rRNA gene sequence-based maximum likelihood phylogenetic tree was calculated from the same alignment with FastTree 2.2^57^employing the GTR + CAT model and 1000 bootstraps replications. Three 16S rRNA gene sequences of bacterial strains from the PVC (*Planctomycetota-Verrucomicrobiota-Chlamydiota* ) superphylum outside of the phylum *Planctomycetota*, namely *Opitutus terrae* (NCBI acc. no. AJ229235), *Kiritimatiella glycovorans* (acc. no. NR_146840) and *Lentisphaera araneosa* (acc. no. NR_027571), were used as outgroup. The multi-locus sequence analysis (MLSA)-based phylogenetic tree was calculated using autoMLST with 500 bootstrap replicates^58^. The analysis was performed with the autoMLST-simplified-wrapper tool available on GitHub. It included all reference genomes of strains belonging to the current family *Planctomycetaceae* and the genomes of *Rhodopirellula baltica* SH1^T^(GenBank acc. no. BX119912.1), *Pirellula staleyi* DSM 6068^T^(acc. no. CP001848.1) and *Blastopirellula marina* DSM 3645^T^(acc. no. GCA_000153105.1) (all belonging to the family *Pirellulaceae* ) served as outgroup. Average amino acid identities (AAI) and average nucleotide identities (ANI) were calculated using the scripts provided in the enveomics collection^59^. The percentage of conserved proteins (POCP) and sequence identities of a partial sequence of the *rpoB* gene encoding the β-subunit of RNA polymerase were calculated as described^60,61^.

### Analysis of genome-encoded features

Estimation of the metabolism and pangenome reconstruction based on the genome sequences of the selected strains were performed with anvi’o v.8^62^. The analyses were conducted with standard parameters based on workflows provided on the anvi’o website (Metabolism suite: https://anvio.org/tutorials/fmt-mag-metabolism, pangenomics: https://merenlab.org/2016/11/08/pangenomics-v2). Secondary metabolite-associated biosynthetic gene clusters (BGCs) were predicted with antiSMASH bacterial version 8.0^63^with relaxed strictness and all extra features enabled. Carbohydrate-active enzymes (CAZymes) were determined with dbCAN3^64^. The analysis was performed in the prokaryotic mode with Pyrodigal as open reading frame finder, DIAMOND for annotation and pyhmmer for sequence analysis against the dbCAN-HMM and dbCAN-sub-HMM databases.

### Nucleotide sequence accession numbers

The 16S rRNA gene sequences were deposited in the GenBank database under the accession numbers PV956138 (SH203), PV956150 (SH248^T^) and PV956165 (SH280). The 16S rRNA sequence of strain SH248^T^has previously been deposited under the acc. no. AJ231186.1^65^. Due to identical 16S rRNA gene sequences of the three strains, only the genome of strain SH248^T^was sequenced. Its genome sequence was deposited under NCBI acc. no. CP197413.

## Results and discussion

### Phylogenetic inference

**Fig. 1:**
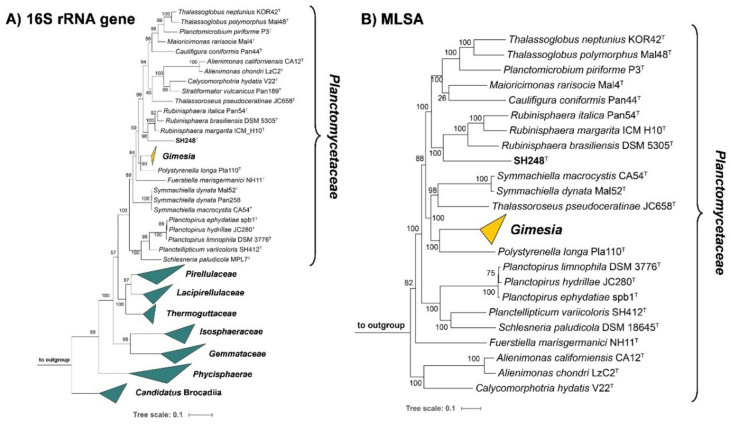
Maximum likelihood phylogenic trees. 16S rRNA gene sequence-based (**A** ) and MLSA-based phylogenies (**B** ) highlighting the position of strain SH248^T^in relation to its closest relatives. Bootstrap values after 1000 (16S rRNA gene sequence-based tree) or 500 re-samplings (MLSA-based tree) are given at the nodes (%). The scale bars indicate the number of substitutions per position.

**Fig. 2:**
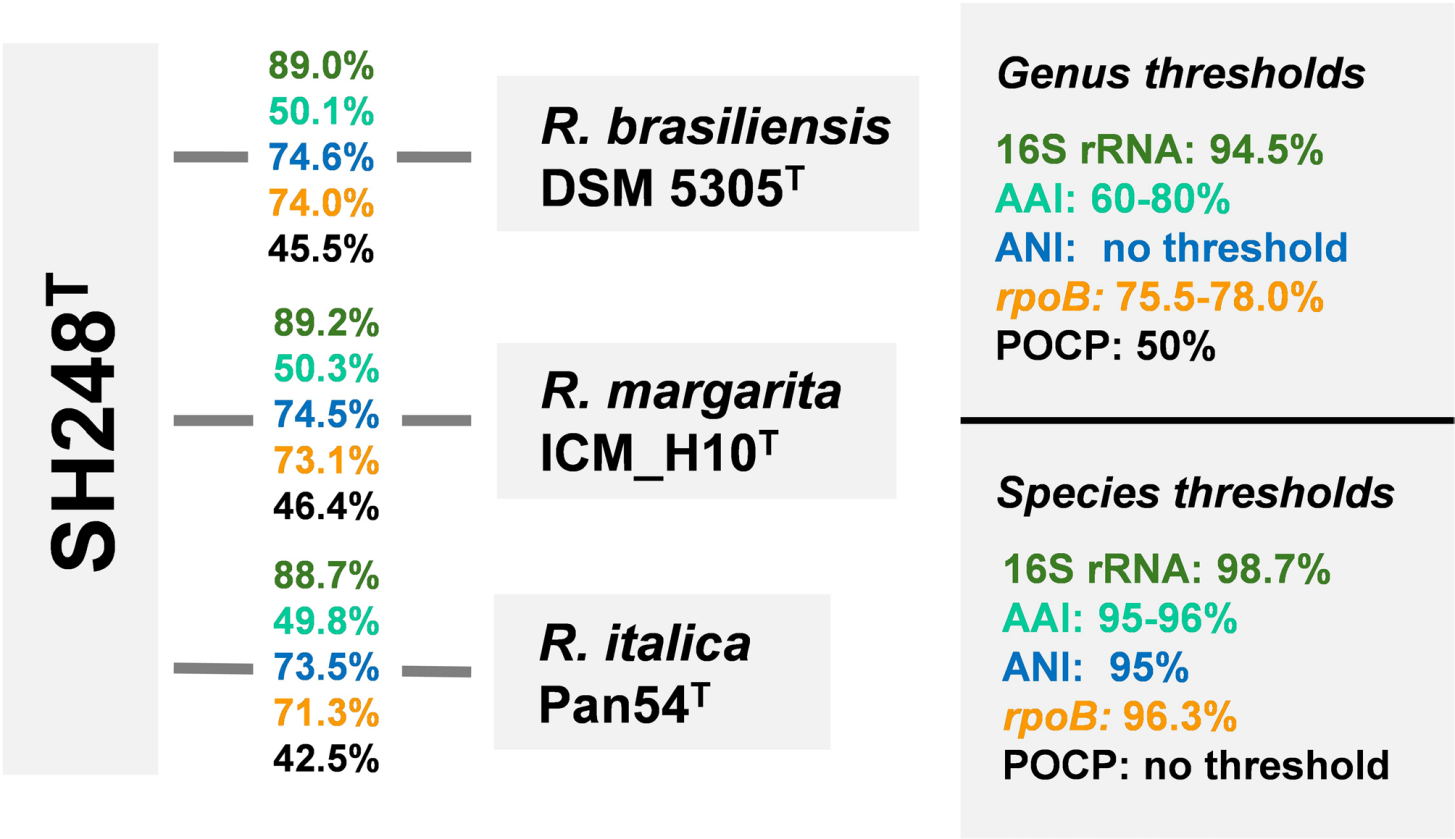
Phylogenetic markers. Comparison of strain SH248^T^to its closest relatives in the genus *Rubinisphaera*. Markers used include 16S rRNA gene sequence identity (16S rRNA), average amino acid identity (AAI), average nucleotide identity (ANI), partial *rpoB* gene sequence similarity and percentage of conserved proteins (POCP).

The comparison of the 16S rRNA gene sequence of strains SH203, SH248^T^, and SH280 yielded identical sequences, indicating a close relationship of the strains on the level of the same species (98.7% species threshold)^66^. In both phylogenetic trees, the strain(s) clustered in the family *Planctomycetaceae* and next to the described members of the genus *Rubinisphaera* (Fig. 1). The genus *Rubinisphaera* constitutes three described species, (1) *Rubinisphaera brasiliensis* DSM 5305^T^that was originally isolated from a water sample from Lagoa Vermelha in Brazil^67^, (2) *Rubinisphaera italica* Pan54^T^isolated from an alga from a hydrothermal area on Panarea Island, Italy^68^, and (3) *Rubinisphaera margarita* ICM_H10^T^isolated from marine sediments of Matosinhos beach in Portugal^69^. A nucleotide BLAST analysis of the full-length 16S rRNA gene sequence of strain SH248^T^yielded a sequence identity below 90% to either of the type strains of the *Rubinisphaera* species (Fig. 2). These values fall far below the genus threshold of 94.5%^66,70^, supporting the assignment of strain SH248^T^to a novel species of a separate genus. The comparison of a 1298 bp partial sequence of *rpoB* between strain SH248^T^and the type strains of *R. margarita*, *R. brasiliensis*, and *R. italica* yielded identity values between of 71 and 74% which fall below the 75.5%-78.0% genus boundary^71^. Similar results were also obtained for the other examined phylogenetic markers (Fig. 2). The values consistently fell below the respective threshold for the delineation of genera for AAI (60%-80%)^72^, and POCP (50%)^60^. ANI values in the range of 73–75% exclude that strain SH248^T^belongs to any known species (species threshold of 95%)^73^(Fig. 2).

### Genomic characteristics

The genome of strain SH248^T^has a length of 5.4 Mbp and is smaller than that of its closest relatives *R. brasiliensis* DSM 5305^T^(6.0 Mbp), *R. margarita* ICM_H10^T^(5.9 Mbp), *and R. italica* Pan54^T^(6.7 Mbp) (Table 1). The DNA G + C content of 54.6% is slightly higher than that of the type strain of *R. italica* (48.8%) but lower compared to type strains of *R. brasiliensis* (56.4%), and *R. margarita* (58.0%). The genome of strain SH248^T^has a coding density of 85.2%. The automated gene annotation using NCBI’s Prokaryotic Genome Annotation Pipeline (PGAP) identified 4,369 genes which corresponds to about 806 genes per Mbp. Of the identified genes, 4,308 are annotated as protein-coding genes with about 1,060 (24.6%) encoding hypothetical proteins. The genome contains 43 tRNA genes and a single copy each of 5S, 16S, and 23S rRNA genes. Genome mining using antiSMASH yielded eight BGCs with the majority being involved in the biosynthesis of terpenoids or relevant precursors. In the *Rubinisphaera* spp. BGCs related to terpenoid biosynthesis were also predominant (Table 2). Strain SH248^T^has an additional cluster containing a putative type I polyketide synthase-encoding gene, but lacks clusters involved in the biosynthesis of the compatible solute ectoine and of an uncharacterized ribosomally-synthesized peptide that are conserved in all three *Rubinisphaera* species. CAZyme profiles or the four compared strains are similar, with most hits in the classes of glycoside hydrolases and glycosyltransferases and between 20 and 25 CAZyme-encoding genes per Mbp (Table 2).

**Table 1:** Phenotypic and genotypic features of SH248^T^in comparison to its current closest relatives of the genus *Rubinisphaera*.

Characteristics	SH248^T^	*Rubinisphaera brasiliensis* DSM 5305^T^	*Rubinisphaera margarita* ICM_H10^T^	*Rubinisphaera italica* Pan54^T^
Phenotypic features				
Isolation location	Campus pond, University of Kiel, Germany.	Lagoa Vermelha, Brazil (surface water)	Marine sediments (Portugal)	Alga from a hydrothermal area (Italy)
Shape	pear-shaped	spherical to ovoid	spherical to ovoid	pear-shaped
Length x width (µm)	2.0 × 1.0	1.8 × 0.7	1.7 ± 0.3 × 1.5 ± 0.3	1.6 × 0.8
Colour	White	Yellow to orange	Cream to beige	White
Relation to oxygen	Strictly aerobic	Aerobic, capable of microaerobic growth	Aerobic, capable of microaerobic growth	n.a.
Temperature range (optimum) (°C)	18–32 (28)	27–35	20–37 (20–30)	14–27 (26)
pH range (optimum)	6.0–10.0 (7.0)	n.a.	6.0–9.0 (6.5–7.0)	6.0–10.0 (9.0)
Doubling time (h)	8	n.a.	40	18
Division	Budding	Budding	Budding, tubular structure connecting the mother and daughter cell	Budding
Budding mode	Polar	Polar	Polar	Polar
Bud shape	Like mother cell	Like mother cell	n.a.	Like mother cell
Dimorphic life cycle	n.o.	Yes	n.a.	n.o.
Motility	Yes	Yes	n.a.	n.a.
Aggregates	No	Yes	Yes	Yes
Genomic features				
Genome size (bp)	5,422,702	6,006,602	5,979,725	6,704,479
Number of contigs	1 (closed)	1 (closed)	22	4
Plasmids	No	No	n.o.	n.o.
DNA G + C (%)	54.6	56.4	58.0	48.8
Coding density (%)	85.2	86.1	86.7	85.2
Genes (total)	4,369	4,706	4,565	5,208
Genes per Mbp	806	783	763	777
Protein-coding genes	4,308	4,625	4,489	5,108
Protein-coding genes per Mbp	795	770	751	762
Hypothetical proteins*	1,060	1,028	991	1288
Hypothetical proteins (%)	24.6	22.2	22.1	25.2
rRNA genes (5S, 16S, 23S)	1,1,1	2,2,2	1,1,1	1,2,1
tRNAs	43	46	51	53

n.o. not observed, n.a. not analyzed. *based on the RefSeq-annotated genomes.

**Table 2:** Genome-based analysis of CAZyme (Carbohydrate-active enzyme) profiles and secondary metabolite biosynthetic gene clusters of strain SH248^T^in comparison to its closest relatives of the genus *Rubinisphaera.*

Characteristics	SH248^T^	*Rubinisphaera * *brasiliensis* DSM 5305^T^	*Rubinisphaera* *margarita* ICM_H10^T^	*Rubinisphaera * *italica* Pan54^T^
Carbohydrate-active enzymes				
Glycoside hydrolases	40	42	57	45
Glycosyl transferases	51	71	54	53
Polysaccharide lyase	1	3	4	2
Carbohydrate esterases	11	18	19	19
Carbohydrate-bind. Modules	10	12	11	14
Auxiliary activities	3	2	3	1
Total number of CAZyme genes	116	148	148	134
CAZyme genes per Mbp	21	25	25	20
Biosynthetic gene clusters				
Terpenoid (incl. precursor)	6	6	7	7
Type I PKS	1	0	0	0
Type III PKS	1	0	1	1
Mixed type I PKS-NRPS	0	1	0	0
Class II lanthipeptide	0	1	0	0
Unspecified ribosomally synthesized peptide product	0	1	1	1
Resorcinol	0	0	1	0
Ectoine	0	1	1	1
Total number of BGCs	8	10	11	10
BGCs per Mbp	1.5	1.7	1.8	1.5

**Fig. 3:**
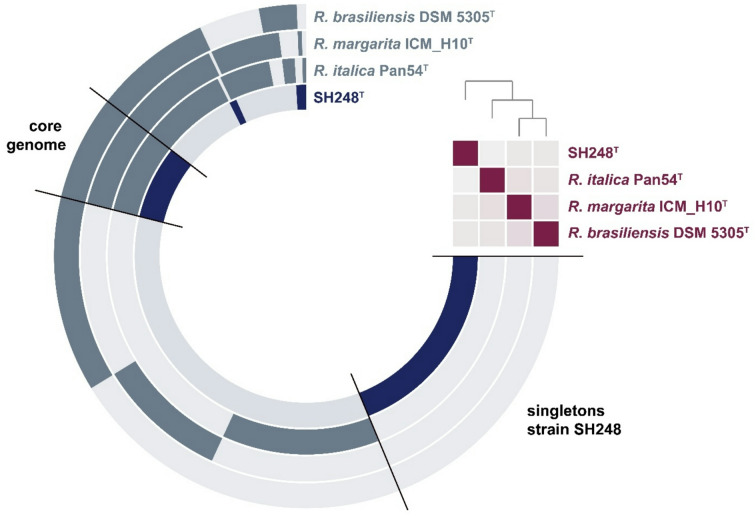
Pangenome of strain SH248^T^and its closest relatives in the genus *Rubinisphaera*. Each open circle represents the pangenome of all strains but is colored darker when the gene is present in the respective genome. The matrix in the upper right corner indicates the degree of relationship of the strains according to average nucleotide identity values (faint colour: ANI ≤ 70%, bright color: ANI = 100%). Singletons of strain SH248T (Table S1) and clusters representing the core genome of the four analyzed genomes are labelled.

The pangenome of the compared strains consists of 11,465 gene clusters (Fig. 3), of which 1,051 are conserved in all four genomes (core genes). The anvi’o pipeline identified 2,890 genes as singletons of strain SH248^T^of which 1,592 could be assigned a (predicted) function (putative annotation). The list of proteins encoded by singletons of strain SH248^T^(Table S1) includes DNA/RNA-modifying enzymes, transporters and components of secretion systems, transcriptional regulators and specialized sigma factors, protein kinases, outer membrane proteins, phage-related proteins and mobile elements, toxin-antitoxin systems and several others. The core genome was not analyzed in further detail, but may help to identify currently uncharacterized essential genes in future studies, e.g. those involved in the planctomycetal cell division by budding. The genome-based analysis of complete metabolic pathways using the “Estimate metabolism” workflow of anvi’o did not point towards major differences between strain SH248^T^and the type strains of the three described *Rubinisphaera* species (Table S2). Central pathways of the carbon metabolism are complete in all four strains. The same is true for anabolic pathways involved in the biosynthesis of sugars (gluconeogenesis), amino acids, fatty acids, nucleotides, vitamins and cofactors. As also suggested by antiSMASH, strain SH248^T^lacks biosynthetic enzymes for the compatible solute ectoine that are conserved in *Rubinisphaera* spp. Minor differences were found in the presence of pathways related to the degradation of amino acids, fatty acids (β-oxidation) and ribonucleotides.

### Morphological, physiological, and biochemical analyses

**Fig. 4:**
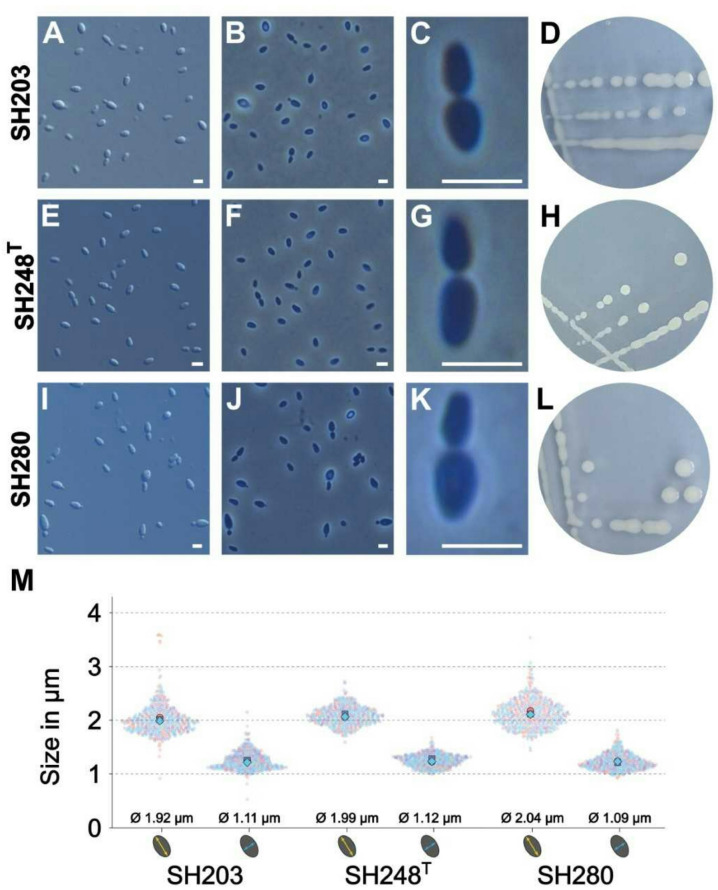
Colony and cell morphology of the novel isolates. Cell morphology and cell division of the isolates were analyzed using differential interference contrast (**A**,**E**,**I** ) and phase contrast microscopy (**B**,**C**,**F**,**G**,**J**,**K** ). The scale bars represent 2 μm. Photographs of agar plates visualizing colony morphology and color of the investigated strains (**D**,**H**,**L** ). Cell sizes were determined from three biological replicates per strain and 150 individual cells per replicate were analyzed (M).

Colonies of strains SH203, SH248^T^, and SH280 are circular, convex and white (Fig. 4), indicating the absence of pigmenting compounds similar to *R. italica* Pan54^T^^68^. The color differs from that of the type strains of the other two *Rubinisphaera* species whose colonies are cream to beige in case of *R. margarita* ICM_H10^T^^69^ and yellow to orange in case of *R. brasiliensis* DSM 5305^T^^67^. Cells of all three strains, SH203, SH248^T^, and SH280, are pear-shaped, motile and divide by budding (Fig. 4). The daughter cells have the same shape as the mother cells. The measured average cell sizes (length x width) are 1.9 × 1.1 μm (strain SH203), 2.0 × 1.1 μm (strain SH248^T^) and 2.0 × 1.0 μm (strain SH280). The strains do not form aggregates, a characteristic separating them from the rosette- or shapeless aggregate-forming species of the genus *Rubinisphaera* ^67–69^(Table 1).

The novel isolates are mesophilic with growth over a temperature range of 18–30 °C (strain SH203), 18–32 °C (strain SH248^T^) or 21–30 °C (strain SH280), with all three having an optimal temperature for growth of 28 °C. Only strain SH248^T^grew above 30 °C; thus, the upper temperature limit is lower than that of the type strains of *R. brasiliensis* and *R. margarita* (both still grow at 35–37 °C). The novel isolates are neutrophilic. Extracellular pH values of 6.0–9.0 (strain SH203), 6.0–10.0 (SH248^T^), and 6.0–8.0 (SH280) allowed for growth and pH 7.0 was optimal for all three (Table 1). There was no major difference in the pH profiles of the three strains when compared to their closest relatives in the genus *Rubinisphaera*. The novel isolates are aerobes like their current closest neighbors. However, microaerobic growth was reported for the type strains of *R. brasiliensis* and *R. margarita* ^69,74^. With generation times of 8–11 h the novel isolates belong to the faster-growing planctomycetes, not only compared to the *Rubinisphaera* species, but also compared to several other members of the family *Planctomycetaceae* (typical division times of 7–70 h). Strain SH248^T^reached a maximal growth rate of 0.087 h^­1^at the optimum temperature and pH which is equivalent to a generation time of 8 h. In the tested medium that contains yeast extract and peptone, supplementation of vitamin B_12_was not essential for growth of strain SH248^T^, but its supplementation improved biomass formation. Like the three *Rubinisphaera* species, strain SH248^T^harbors the heterodimeric vitamin B_12_-independent ribonucleotide reductase NrdAB. This enzyme belongs to class I of ribonucleotide reductases that use a di-iron-tyrosyl radical cofactor instead of vitamin B_12_^75^. In contrast, the limnic model planctomycete *P. limnophila* relies on the vitamin B_12_-dependent class II enzyme NrdJ and is thus strictly dependent on the supplementation of vitamin B_12_. Since the medium contains complex ingredients including amino acids, a potential dependence on vitamin B_12_for the biosynthesis of methionine in strain SH248^T^is not phenotypically visible under the chosen cultivation conditions.

### Ecological and environmental context of the novel taxon

A nucleotide BLAST analysis of the 16S rRNA gene sequence of strain SH248^T^against NCBI‘s nucleotide database yielded only a single sequence of an uncharacterized strain belonging to the same species and genus (99.2% similarity, acc. no. JX307094.1). The sequence belongs to a strain detected in oil well water in Xinjiang, China. In the GTDB taxonomy, the novel isolate belongs to the uncharacterized genus and species listed under placeholders SXPC01 and sp005778225, respectively. The GTDB genus representative is GenBank acc. no. GCA_041753175.1, a metagenome-assembled genome (MAG) reconstructed from a biofilm sample from the arctic geothermal Jotun spring in Svalbard (Spitsbergen), Norway. The GTDB species representative is GenBank acc. no. GCA_005778225.1, a freshwater MAG from Fuxian Lake, Yuxi, China. Since both MAGs lack 16S rRNA gene sequence information, ANI was used instead. ANI values of 77.0% and 92.7% were obtained for the comparison of strain SH248^T^with GCA_041753175.1 and GCA_005778225.1, respectively, indicating that neither of the strains represented by the two MAGs belongs to the same species as strain SH248^T^(95% species threshold). An analysis of POCP yielded values of 57.6% and 79.7% for the same genome combinations, respectively, supporting the assignment to the same genus (50% genus threshold).

## Conclusion

Analysis of the collected data on phenotypic and genomic characteristics led to the conclusion that the three strains SH203, SH248^T^and SH280 represent a joint novel species of a novel genus in the family *Planctomycetaceae*. We therefore introduce the name *Lacunimicrobium album* gen. nov., sp. nov. for the novel taxon. Strain SH248^T^is the type strain of the novel species.

### Description of *Lacunimicrobium* gen. nov.

La.cu.ni.mi.cro’bi.um. L. fem. n. *lacuna*, a little lake, N.L. neut. n. *microbium*, a microbe; N.L. neut. n. *Lacunimicrobium*, a microbe isolated from a lake.

Members of the genus form pear-shaped, motile cells that divide by budding. Colonies are circular, convex and non-pigmented. Cells are obligate aerobic heterotrophs with a mesophilic and neutrophilic growth profile. The DNA G + C content is around 55%. The genus belongs to the family *Planctomycetaceae*, order *Planctomycetales*, class *Planctomycetia*, phylum *Planctomycetota*. The type species is *Lacunimicrobium album*.

### Description of *Lacunimicrobium album* sp. nov.

al’bum. L. neut. adj. *album*, white, referring to the color of the colonies.

In addition to the features described for the genus, the species is characterized by the following properties: Cells have an average length and width of 2.0 ± 0.2 × 1.0 ± 0.2 μm. Daughter cells have the same shape as mother cells. Cells do not form macroscopic or microscopic aggregates. The type strain is SH248^T^(= CECT 30703^T^ = STH01007^T^, the STH number refers to the Jena Microbial Resource Collection JMRC) and was isolated from a campus pond at Kiel University in Northern Germany. The type strain grows over a temperature range of 18–32 °C and a pH range of 6.0–10.0 and shows optimal growth at 28 °C and pH 7.0. In the tested medium, a generation time of 8 h was observed during the exponential growth phase and the supplementation of vitamins was not required for growth. The type strain genome has a size of 5.42 Mbp and a DNA G + C content of 54.6% and lacks extrachromosomal elements. Non-type strains belonging to the species are SH203 (= CECT 30704 = STH01006) and SH280 (= CECT 30705 = STH01008).

## Supplementary Information

Below is the link to the electronic supplementary material.

Supplementary Material 1

Supplementary Material 2

## Data Availability

The 16S rRNA gene sequences of the novel isolates were deposited in the GenBank database under the accession numbers PV956138 (SH203), PV956150 (SH248^T^) and PV956165 (SH280). The genome sequence of strain SH248^T^was deposited under NCBI acc. no. CP197413.
